# Core–Shell Nanorods as Ultraviolet Light-Emitting Diodes

**DOI:** 10.1021/acs.nanolett.2c04826

**Published:** 2023-02-07

**Authors:** Douglas Cameron, Pierre-Marie Coulon, Simon Fairclough, Gunnar Kusch, Paul R. Edwards, Norman Susilo, Tim Wernicke, Michael Kneissl, Rachel A. Oliver, Philip A. Shields, Robert W. Martin

**Affiliations:** † Department of Physics, Scottish Universities Physics Alliance (SUPA), University of Strathclyde, Glasgow G4 0NG, United Kingdom; ‡ Department of Electrical & Electronic Engineering, University of Bath, Bath BA2 7AY, United Kingdom; § Centre de Recherche sur l’Hétéro-Epitaxie et ses Applications (CRHEA)−Centre National de la Recherche Scientifique (CNRS), Rue Bernard Grégory, 06560 Valbonne, France; ∥ Department of Materials Science and Metallurgy, University of Cambridge, CB3 OFS Cambridge, United Kingdom; ⊥ Institute of Solid State Physics, Technische Universität Berlin, Hardenbergstraße 36, 10623 Berlin, Germany

**Keywords:** UV LED, nanowire, core−shell, AlGaN, semiconductor, electron microscopy

## Abstract

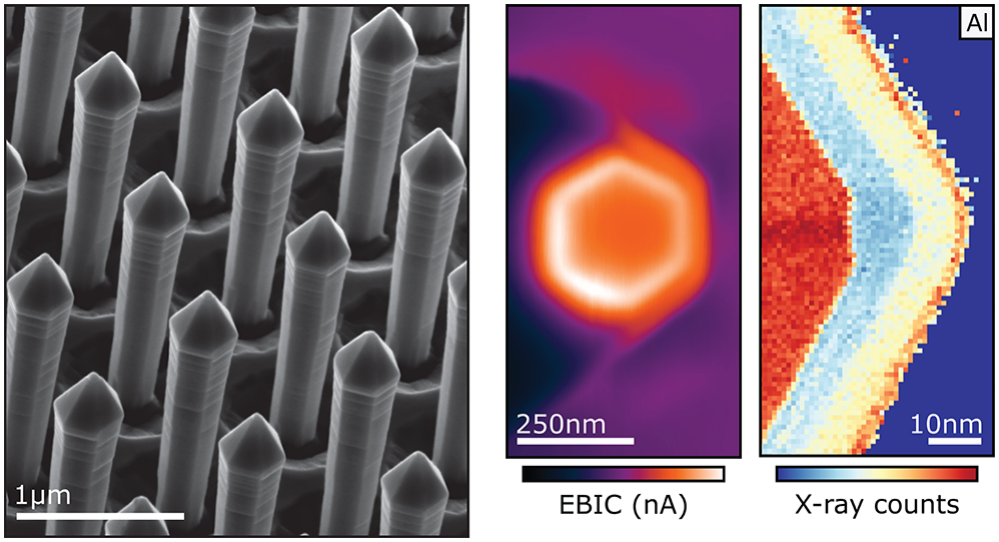

Existing barriers to efficient deep ultraviolet (UV) light-emitting diodes (LEDs) may be reduced or overcome by moving away from conventional planar growth and toward three-dimensional nanostructuring. Nanorods have the potential for enhanced doping, reduced dislocation densities, improved light extraction efficiency, and quantum wells free from the quantum-confined Stark effect. Here, we demonstrate a hybrid top-down/bottom-up approach to creating highly uniform AlGaN core–shell nanorods on sapphire repeatable on wafer scales. Our GaN-free design avoids self-absorption of the quantum well emission while preserving electrical functionality. The effective junctions formed by doping of both the n-type cores and p-type caps were studied using nanoprobing experiments, where we find low turn-on voltages, strongly rectifying behaviors and significant electron-beam-induced currents. Time-resolved cathodoluminescence measurements find short carrier liftetimes consistent with reduced polarization fields. Our results show nanostructuring to be a promising route to deep-UV-emitting LEDs, achievable using commercially compatible methods.

Over the past few decades, III-nitride light-emitting diodes (LEDs) have revolutionized visible lighting, forming remarkably efficient and compact devices. However, the material system offers further potential, including tunable emission from the infrared to the deep ultraviolet (UV).^1,2^ UV light emitters have a multitude of motivating applications, such as water purification,^3^ skin-safe disinfection,^4,5^ and the curing of resins.^6^ By increasing the AlN content in AlGaN-based LEDs, we open a pathway to deep UV emission (down to 205 nm), but performance is currently hampered by a compounding array of deleterious factors.

Conventional III-nitride LEDs are grown as polar *c*-plane layers, in the stable wurtzite crystal structure. The resulting quantum wells contain a high degree of spontaneous and piezoelectric polarization, and through the quantum-confined Stark effect (QCSE), these strong electric fields separate electron and hole wave functions and lower the recombination efficiency.^7^ The growth of nanostructures, such as nanorods, allows for active regions to instead be deposited radially on nonpolar *m* or *a* planes, circumventing the QCSE and improving internal quantum efficiency.^8^

The doping of high AlN content AlGaN poses another challenge, with the high activation energy and, hence, low hole density of Mg dopants consistently producing poor conductivity. For this reason, p-GaN contact layers are commonly used in UV LED structures, resulting in the counterproductive absorption of light generated in the active regions. Surface-doping enhancement in core–shell structures may help alleviate this problem and avoid the use of GaN.^9−11^

High AlN content material also typically displays significant densities of threading dislocations, which can cause current leakage and/or act as non-radiative recombination centers.^12^ Nanowires created through both top-down etching and bottom-up growth methods have been shown to reduce threading dislocation densities through filtering and bending.^13−18^

One final material challenge to mention arises from the two distinct valence band structures of GaN and AlN. These affect the optical polarization from AlGaN quantum wells, with AlN-rich alloys having strong emission perpendicular to the *c* axis. Light extraction from the top surface of *c*-plane quantum wells therefore becomes problematic. Although, with *m*-plane AlGaN on AlGaN quantum wells, the polarization of emission will also be perpendicular to the *c* axis,^19^ with nanorods, the light can escape the surface with greater ease and even be preferentially redirected along the *c* direction with some specific configuration of the array (e.g., pitch, height, and diameter).^20,21^ Core–shell nanorods will also have greater quantum well and junction areas relative to their footprint, and this can improve current spreading and reduce the efficiency droop at high current densities.^22−24^

To unlock the full potential of such technology, advanced production techniques must be adopted to create regular and well-defined nanostructures. Whereas the selective area growth of GaN nanorod core arrays and the subsequent growth of AlGaN and InGaN shells have already been demonstrated by metal organic vapor phase epitaxy (MOVPE),^25^ such growth of AlN and AlGaN rods remains elusive as a result of the very high sticking coefficient and the low diffusion length of Al atoms.

Currently, most AlGaN nanorods are grown by molecular beam epitaxy (MBE), which possesses a limited throughput in comparison to MOVPE. Furthermore, MBE AlGaN-based nanorods typically require a GaN pedestal grown on silicon to initiate their nucleation. The narrower band gaps of these materials result in the detrimental absorption of light and lower the external quantum efficiencies of devices.

A solution to this problem is the combination of top-down etching to form a uniform array of nanorod cores followed by MOVPE overgrowth of the active material.^26^ This approach maintains the benefits of conventional core–shell structures while introducing additional design flexibility. The core material can be formed from a range of two-dimensional (2D) planar materials, including ternary or quaternary alloys, and the configuration of the nanorod arrays can be tuned through advanced patterning techniques and well-controlled top-down etching.

With this hybrid approach, we recently demonstrated the synthesis of highly uniform and organized AlN nanorods on sapphire substrates^27^ and the successful fabrication of deep UV AlN/AlGaN core–shell structures.^28^ However, as a result of the increased ionization energy of the Si donor combined with self-compensation effects, even Si-doped AlN is found to be highly resistive.^29^ To achieve electrical injection, it is necessary to create a n-doped core with a reasonably low resistivity upon which subsequent quantum wells (QWs) and then p-AlGaN can be grown; thus, an alternative to AlN must be employed for the core.

In this work, we report on the fabrication and characterization of highly uniform AlGaN core–shell nanorod LED structures complete with p–n junctions. Our structure combines etched n-AlGaN cores with the MOVPE overgrowth of an AlGaN QW and a p-AlGaN capping layer. This design improves lattice matching between layers and allows for better light extraction while still maintaining the high conductivity required for an effective electrically driven device. The UV-transparent sapphire substrate also enables backside light extraction, impossible with alternative candidates such as GaN or Si.^30−32^

## Assembly of AlGaN Core–Shell Structures

First, we grew a 3 μm thick n-Al_0.76_Ga_0.24_N layer atop an AlN/sapphire template by MOVPE. This layer had a doping level of 2.1 × 10^19^ cm^–3^ and resistivity of 0.04 Ω cm.

We used displacement Talbot lithography^33,34^ and a lift-off process to define a metal dot mask on top of n-AlGaN. An inductively coupled plasma etch followed by a KOH wet etch then shaped our nanorod cores to diameters of 210 nm and heights of 1.7 μm.^27,28^

**Figure 1:**
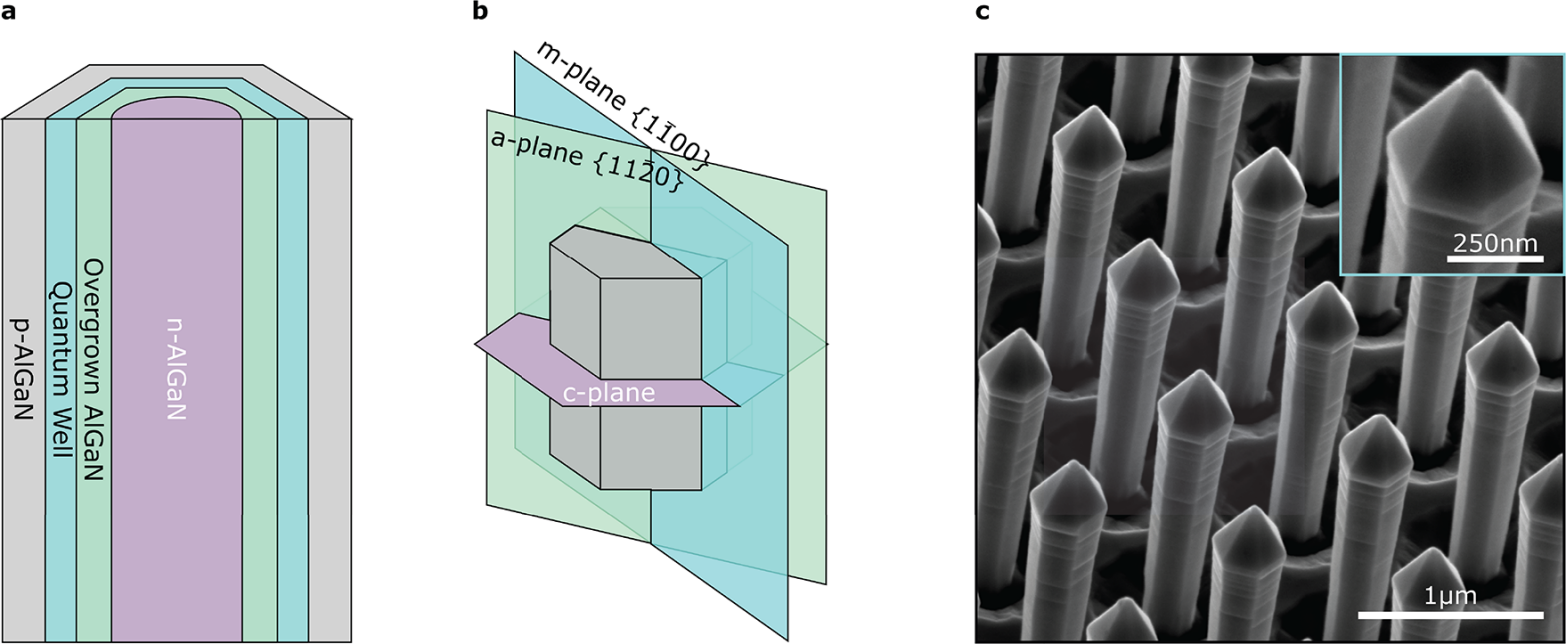
Nanorod core–shell architecture. (a) Schematic of core–shell structures employed in this work (not to scale), with n-AlGaN cores, quantum wells, and p-AlGaN shells forming a full LED structure. (b) Frequently discussed crystal planes relating to our rods, which also identify the orientation of our TEM lamellae. (c) SE image of the nanorod array with clear uniformity in pitch and rod dimensions. Scale bar is 1 μm. A higher magnification inset (with a 250 nm scale bar) shows a spherical feature at the tip of a rod.

Initiating MOVPE overgrowth on these n-type cores with a preliminary AlGaN layer recovered well-defined nonpolar and semipolar facets, over which we deposited a thick single quantum well and a Mg-doped p-AlGaN cap. Our full structure is depicted in Figure 1a.

We intend the lowest band gap layer in our structure to be the quantum well, avoiding any unnecessary light absorption from surrounding layers. The high uniformity and morphology of our structures as a result of this hybrid method are evidenced in Figure 1c.

## Composition and Structure

Using transmission electron microscopy (TEM) and energy-dispersive X-ray spectroscopy (EDS), we investigated the composition and structure of our nanorods. We prepared two suitably electron transparent lamellae in the focused ion beam (FIB) microscope to examine rods in two of the planes visualized in Figure 1b. The first section was along the *c* plane near the top of the nanorods, just below where the semipolar and nonpolar facets intersect. The second section ran the length of the rods along the bisecting *m* plane.

**Figure 2:**
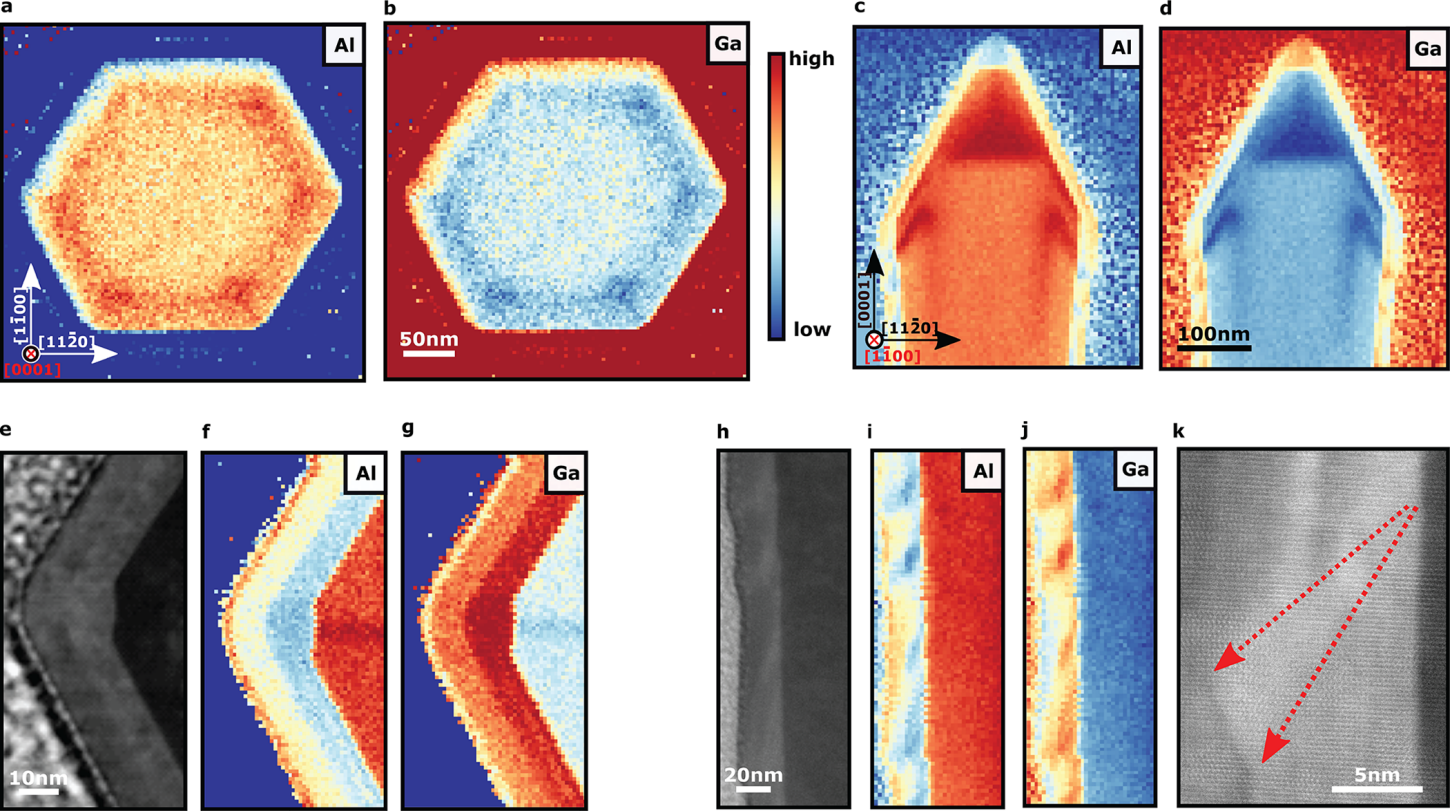
TEM–EDS elemental composition maps. (a) Low-magnification map of the first section looking along the *c* direction. The Al X-ray peak intensity variation within the core reveals increased Al incorporation on the *a* axes. (b) Ga K- and L-line X-ray peak intensities within the core showing the inverse of the Al map as expected. (c and d) Low-magnification map of the second section looking through the *m* direction. The formation of the “broadhead” can be seen here. (e) Higher magnification high-angle annular dark-field (HAADF) image focusing at one of the corners of the hexagonal structure. *Z* contrast in the images reveals the location of the core, quantum well, and p-AlGaN capping layer. The formation of a distinct *a* plane at the edge of the core is clear. (f) Al X-ray peak intensity showing both the increased Al incorporation when moving from the core center along the *a* direction and decreased Al in the quantum well on the *a*-plane facet. (g) Ga X-ray peak intensity with the core again showing the inverse of the Al map as expected. (h, i, and j) HAADF and Al and Ga X-ray peak intensities over the same area, showing alloy fluctuations along the quantum well. The Ga-rich composition develops in a semipolar direction from the *a* plane. (k) Higher magnification HAADF TEM image focusing along one of the *a* planes. Here, we can see the exacerbation of surface roughness as growth progresses outward.

Panels a and b of Figure 2 focus on the initial overgrowth via the *c*-plane section. Here, the contrast in the EDS maps has been expanded to highlight a difference in composition between the n-AlGaN etched core and the AlGaN regrowth layer, with the later being richer in Al. In addition, higher AlN incorporation is observed along the [112̅0] direction normal to the *a* plane.

From the same lamella, we also observe the formation of distinct *m* and (smaller) *a* facets as seen in panels e, f, and g of Figure 2. The *a* planes are most distinct where the single quantum well growth was initiated and lose their definition as the quantum well develops. This can be explained through growth rate disparities on different facets competing toward the extinction of the faster growing *a* plane. In this case, preferential incorporation of Ga on these planes contributes to thickness and compositional variations in the quantum well, with thicknesses of 7 nm on the *m*-plane facet versus 12 nm on the *a* plane. In contrast, the regrown AlGaN grown prior to the QW exhibits a lower GaN content at the *a* facet than on the main *m* facet.

Previous microscopy of InGaN^35,36^ and hexagonal InGaAlAs^37^ nanorods found similar compositional fluctuations. It is suggested that inhomogeneous strain relaxation within the rod structures is the root cause.^38^ In the structures investigated here, the regrown AlGaN layer has a slightly increased AlN percentage compared to the initial Al_0.76_Ga_0.24_N core and is thus in tension. In that case, to better match the lattice parameter of the core, any excess Al will be preferentially incorporated in the relaxed area. Conversely, the quantum well has a lower composition compared to the regrown AlGaN layer and is thus in compression. As a result, the behavior is now reversed, with excess Ga being preferentially incorporated at the relaxed area to match the lattice of the underlying layer. Differences in the sticking coefficients of Al and Ga ions on the distinct atomic structures of the *a* and *m* planes will also play a role, along with the growth conditions for each layer influencing the favorability of ion incorporation on different facets.^39^

The terminating p-AlGaN capping layer is observed to be more homogeneous than the previous layers as a result of the elimination of *a* planes at this stage, with both the layer thickness and composition fairly uniform. At the surface of the rods, a thin (≈3 nm) heavily oxidized and Al-rich layer appears to form.

The second cut, looking through the *m* plane, reveals multiple complex features. GaN-rich compositional clusters are clear in the quantum wells in panels h, i, and j of Figure 2. We ascribe this effect to the wet etching of the n-AlGaN cores imparting a slight taper with an undercut inclination angle of around 2.00 ± 0.01° and the formation of small surface steps. Through a step-bunching process, these surface steps become larger and more exaggerated as growth progresses. Under the QW growth conditions (higher TMGa flow, lower growth temperature, and higher reactor pressure), these steps become substantial enough to drive significant semipolar growth, resulting in distinct compositional variations in the QWs along the length of the rod.^40,41^ High-resolution TEM images show how small these original protrusions may be while still triggering cluster formation. The enlarged steps are visible on the surface of the rods in panels c and i of Figure 1. Although less pronounced and with a reduced miscut, these steps have previously been observed in GaN/InGaN core–shell nanorods obtained with the same approach.^42^

## Optical Properties

The optical qualities of our structures were first assessed by cathodoluminescence (CL) hyperspectral imaging in an SEM at room temperature.^43^ In addition to the full core–shell LED structure, we prepared examples of Al_0.76_Ga_0.24_N dry/wet etched cores along with rods following the initial facet-recovering overgrowth to explore the systematic effects of these processing steps.

**Figure 3:**
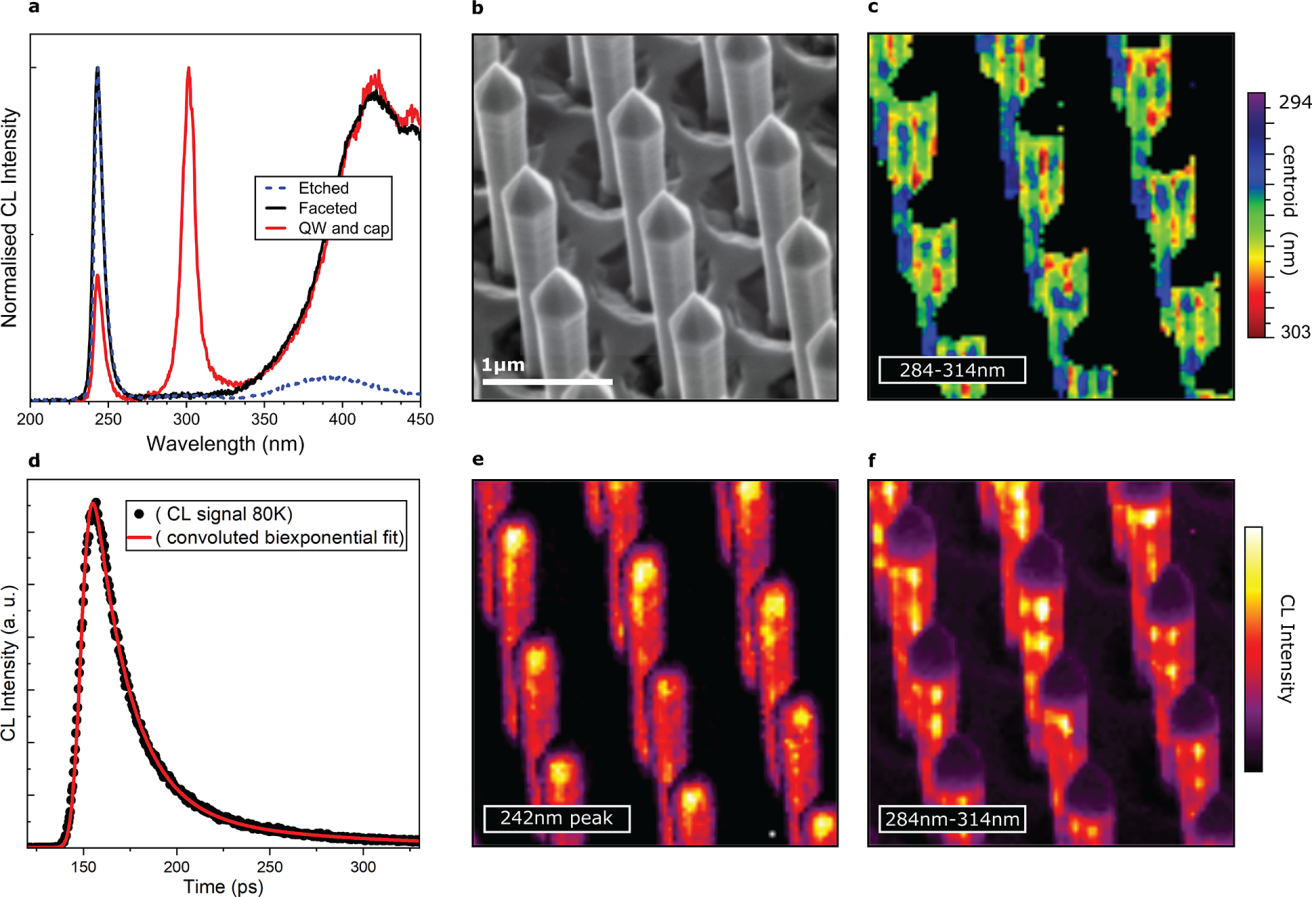
Results from room-temperature, low-temperature, and time-resolved CL hyperspectral studies. (a) CL spectra from the three samples at room temperature: the etched cores, the etched cores following refaceting, and the etched cores following QW and cap growth. Each spectrum was taken by averaging a number of pixels from maps of each sample (taking care to avoid regions where the “substrate” was scanned directly) and was then normalized to a maximum. (b) Secondary electron (SE) image of the area mapped. (c) Map showing the shift in energy of the quantum well emission, with red shifts at the *m*-plane intersections (*a* plane). The noise-dominated substrate region has been masked in this map for clarity. (d) Fitted decay of the *m*-plane quantum well emission accounting for the instrument response function. (e) Map showing the uniform band edge emission peak intensity from the core. (f) CL intensity of the quantum well emission showing distinct high-intensity clusters at the *a* plane along with lower emission intensity from the rest of the *m*-plane sidewalls. Emission from the top semipolar facets is notably absent.

The etched cores display strong band-edge emission at 243 nm and very low defect luminescence (peaking around 392 nm), as seen in Figure 3a. The optical quality of this core is a significant improvement over the AlN core previously employed to create AlN/AlGaN core–shell structures.^28^

The initial overgrowth and faceting step can clearly be seen to introduce a significant point defect population, resulting in multiple luminescence bands in the range of 360–470 nm. These defect bands are ascribed to cation vacancy complexes and are commonly seen in AlGaN alloys.^44−46^ The lack of a band edge emission peak from this layer could be due to the close compositional match to the core combined with the increased defect population. Note that the reactor employed for these overgrowth stages had not been optimized for high-temperature growth, which could explain the high concentration of point defects. Regardless of these initial defective facets, the full core–shell LED structure with a quantum well and p-capping layer was found to be optically active with sharp peaks around 300 nm (fluctuating by around 10 nm). Shorter wavelength emission should be possible using our methods by modifying the quantum well compositions and/or thicknesses.

Multiple overlapping emission peaks from the quantum wells limit the accuracy of spectral peak fitting, and therefore, band-pass maps are a preferable method to show the intensities and centroid energies of different spectral regions, which are shown in Figure 3. The core emission (242 nm) is uniform as expected, with any variation caused by the excitation/collection geometry. Notably, the quantum well emission (284–314 nm) can be seen to be relatively uniform from rod to rod, but along each of these rods, emission is strongest at specific spots on *m*-plane intersections. These correlate to the clusters that we see forming at the internal *a* planes in TEM. A contributing factor to these intensity variations could be the striated surface itself, which may slightly increase the light extraction efficiency and beam energy absorption. The effect of the compositional fluctuations is clear in the centroid map (Figure 3c). Thicker wells would result in a red shift as a result of lower confinement,^47^ as would higher GaN incorporation because the effective band gap of the resultant AlGaN alloy would be lower. We are likely seeing a combination of the two effects but with the significant compositional changes dominating. The high intensity emission of the clusters is likely enhanced through localization effects.

Despite TEM measurements indicating the presence of a thinner (5 nm) quantum well on top of the semipolar facets of the rods, our CL measurements show a notable lack of luminescence from these regions. We suggest this to be due to a significant increase in the population of point defects acting as non-radiative recombination centers incorporated on these secondary facets.^48^ EDS measurements support this interpretation, showing significantly increased oxygen incorporation on these facets and demonstrating the ease with which some impurities may incorporate here.^49^ The radiative recombination rate would also be lower in this plane as a result of the QCSE further attenuating any QW luminescence from this region.

Time-resolved cathodoluminescence (TRCL) measurements find the lifetimes of our *m*-plane transitions to be remarkably short, even at 80 K (in our case, we generally find that lower temperatures extended the carrier lifetimes).^50^ As seen in Figure 3d, a convoluted biexponential decay curve can be fitted to determine a carrier lifetime of 19 ps in the QW while accounting for the instrument response function.^51^ Factors such as localization, well widths, and point defect populations will modify these lifetimes, but such short lifetimes can only be explained by the absence of or significant reduction in the internal electric fields. These short lifetimes are desirable for fast switching devices and may also help to reduce the influence of droop by keeping carrier densities tolerable, allowing for higher optical powers to be extracted.

## Electrical Characterization of p–n Junctions

To check for the successful formation of a p–n junction, we make use of the charge-separating behavior of a depletion region. Electrons and holes generated by the beam will be swept to opposite sides of the junction by any built-in electric field; if the p and n contacts are connected via an external circuit, this will result in a current flow analogous to the photovoltaic effect. The resultant electron-beam-induced current (EBIC) can be used as the image-forming signal for the SEM to map variations in field strength and carrier recombination rates.^52^

**Figure 4:**
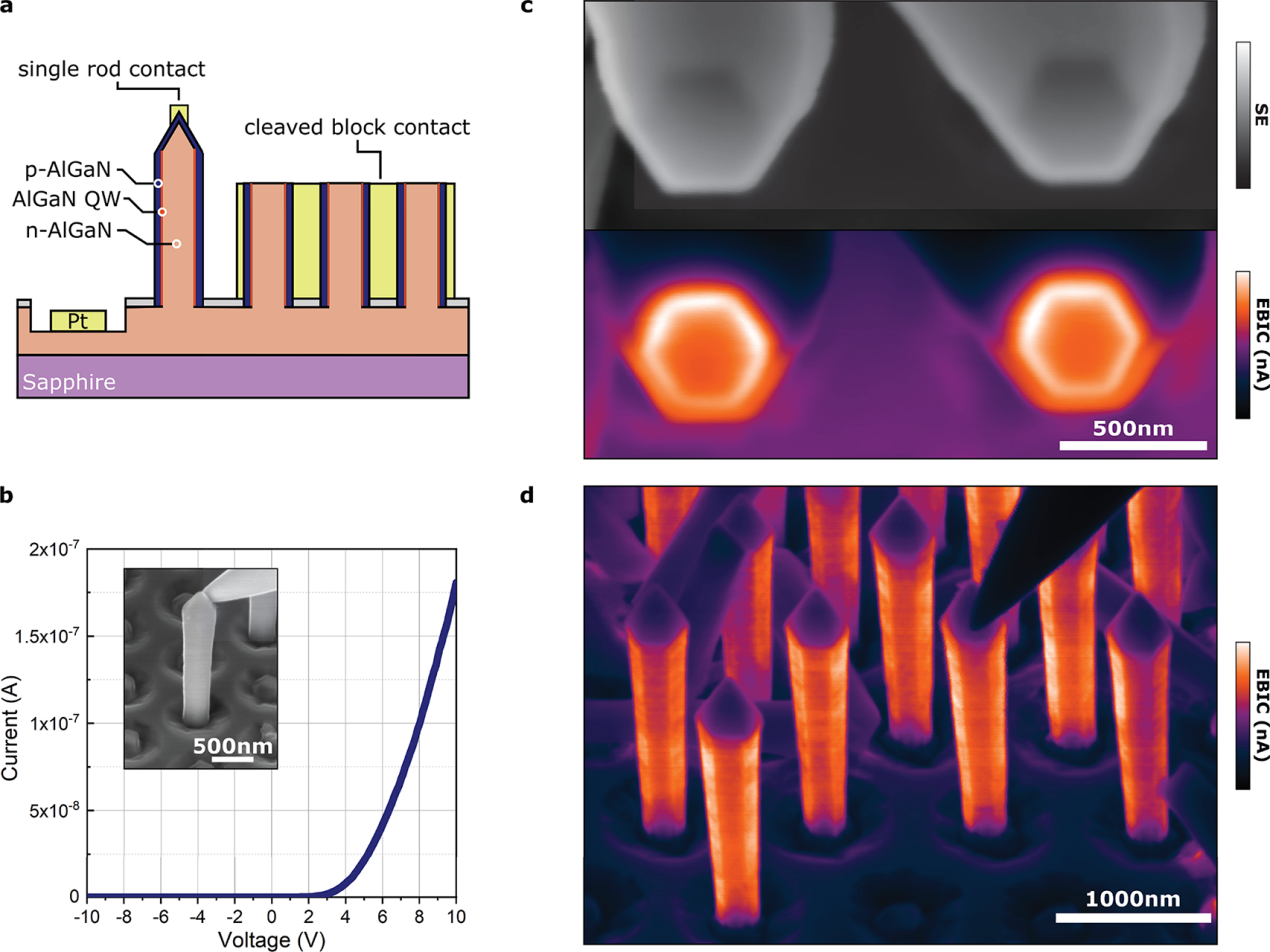
Electrical characterization of our core–shell LED structures. (a) Schematic of our nanorod electrical testing architecture. Using FIB–SEM, a common n contact was created by milling down into the n layer and a Pt pad and then deposited. The p contacts were produced in two forms: Pt was either carefully deposited on the apex of single rods or infilled around many rods to create blocks, which had been cleaved (again using the FIB), to reveal previously obscured inner junctions. (b) Example *I*–*V* curve for a single rod contacted with a nanoprobe as seen in the featured inset. This shows strong rectification, indicating the successful formation of a p–n junction. (c) SE (grayscale) and EBIC images of the cleaved block contact viewed from above, showing the presence of the junction around the entire circumference of the rods as intended. (d) EBIC image of a single-rod contact, with the nanoprobe contacting from above.

For electrical characterization on the single nanorod level, we created two contact schemes in the FIB, both sharing a common n contact, as illustrated in Figure 4a. The first p contact was formed by electron beam deposition of Pt on the tips of individual nanorods. The second involved intentionally cleaving a small array of rods along the *c* plane below the semipolar pyramidal tops and subsequently infilling the array with Pt using Ga-beam deposition.

Measurements on our cleaved block detailed in Figure 4c show a strong uninterrupted p–n junction around all six of the nonpolar *m*-plane sidewalls of each rod. Using our single-rod contacts, we check the quality of these junctions along the length of our rods (Figure 4d). From this perspective, we can see the homogeneity of the junctions, with only some minor contrast variation visible, which relates to the previously described striations.

Analogous to our CL measurements, the pyramidal semipolar facets appear dark in EBIC maps, further indicating low current collection and, therefore, the lack of functional junctions. Similar to the case of CL, non-radiative recombination centers acting as carrier sinks would lower any collected current. The incorporation of compensating impurities during growth or the contact deposition method may additionally contribute to the low EBIC signal here.

Rectification is the defining property of diodes and can be seen in the exponential behavior of our current–voltage (*I*–*V*) curves plotted in Figure 4b. This combined with the EBIC maps confirms not only the existence but also the quality of the junction. Using our single-rod contacts, we performed *I*–*V* sweeps up to ±12 V and found a turn-on voltage around 4.5 V, indicating high doping efficiency and a rectification ratio of 10^5^ post-turn on at ±5 V. We note that the FIB deposition was found to deteriorate the QW luminescence, and for this reason, electroluminescence measurements using our single-wire contacting scheme were not possible.

## Conclusion

We have demonstrated that, through our commercially scalable hybrid top-down/bottom-up method, highly uniform radial core–shell nanorods can be produced from AlGaN with tunable array dimensions. Our “GaN-free” designs upon sapphire substrates prevent self-absorption from lower band gap materials. These effectively transparent device structures could additionally allow for backside light extraction, simplifying future contacting schemes in a commercial device. We confirmed successful doping of our structures and functioning p–n junctions through nanoprobing experiments, where we observed low turn-on voltages (≈4.5 V), high rectification ratios (10^5^ at ±5 V), and substantial electron-beam-induced currents. Short carrier lifetimes (≈19 ps) in the quantum well measured by TRCL are congruent with reduced internal polarization fields. Not only will reduced fields encourage higher efficiency recombination, but they also enable higher device switching speeds not possible in traditional *c*-plane devices. Our TEM measurements do find compositional variations in multiple layers of the heterostructure, which we explain in relation to the initial etching of the n-AlGaN cores. As techniques mature, we envisage the addition of electron blocking layers, multi-quantum well (MQW) structures, and other advancements found to improve conventional LED performance. We expect that the nanostructuring of AlGaN-based LEDs will be key to overcoming the current barriers to efficient deep UV emission in solid state devices.

## Supporting Information Available

Experimental methods for growth, SEM, TEM, and electrical contact formation (PDF)
