# Supplementary material for: Core–Shell Nanorods as Ultraviolet Light-Emitting Diodes
Source: Nano Lett. 2023 Feb 7;23(4):1451–8. doi: 10.1021/acs.nanolett.2c04826 (PMC9951243; doi:10.1021/acs.nanolett.2c04826)
Supplement: Supplementary file 1 — nl2c04826_si_001.pdf [file nl2c04826_si_001.pdf]

Core-shell nanorods as ultraviolet light emitting diodes (S.I.)

Douglas Cameron[1], Pierre-Marie Coulon[2,3], Simon Fairclough[4], Gunnar Kusch[4], Paul R. Edwards[1], Norman Susilo[5], Tim Wernicke[5], Michael Kneissl[5], Rachel A. Oliver[4], Philip A. Shields[2], and Robert W. Martin[1]
[1]*Department of Physics, SUPA, University of Strathclyde, Glasgow G4 0NG, United Kingdom*
[2]*Department of Electrical & Electronic Engineering, University of Bath, Bath BA2 7AY, UK*
[3]*CRHEA-CNRS, Rue Bernard Grégory, 06560 Valbonne, France*
[4]*Department of Materials Science and Metallurgy, University of Cambridge, CB3 OFS Cambridge, United Kingdom and*
[5]*Institute of Solid State Physics, Technische Universität Berlin, Hardenbergstr. 36, 10623 Berlin, Germany*

METHODS

Growth

The *n*-AlGaN template was grown within a close-coupled shower-head MOVPE reactor with standard precursors such as trimethylaluminum (TMAl), trimethylgallium (TMGa), ammonia (NH_3) and silane (SiH_4) as *n*-dopant source. AlN/sapphire templates were overgrown with a 400 nm thick AlN buffer layer followed by a 25 nm AlN to $Al_{0.76}Ga_{0.24}N$ graded transition layer, a 100 nm $Al_{0.76}Ga_{0.24}N$ buffer layer and a 3 μm $Al_{0.76}Ga_{0.24}N$ *n*-doped layer. From this material nanopillars are etched utilizing displacement Talbot lithography and a dry/wet etch as described in the main text. [1]

Atop these pillars, the AlGaN shell is overgrown within a horizontal MOVPE reactor using the standard precursors mentioned above, as well as cyclopentadienylmagnesium (Cp_2Mg) as a *p*-dopant source. The shell stack consists in an undoped AlGaN layer (to recover well defined non-polar lateral and semi-polar top facets), a relatively thick AlGaN quantum well and a *p*-AlGaN doped layer. To ensure activation of the acceptors the completed structures are then annealed at 900°C in a nitrogen atmosphere.

The final nanorods were found to have a length of 2 μm, diameters $\approx$ 350 nm and a pitch of 1.5 μm.

Scanning Electron Microscopy

RTCL was measured using our custom-built system housed within an FEI Quanta 250FEG [2], which can remain functional while nanoprobes are in use. Samples were mounted at 45° to both beam and collection on a pre-tilted stub. Light is collected using a NA0.28 reflecting objective which focusses the light through the 50 μm entrance slit of a 1/8 m Oriel MS125 spectrometer with a 600 lines/mm grating. This spectrometer is paired with a 1600 pixel Andor Newton cooled electron multiplying charge-coupled device detector. For the maps shown in Fig. 3 an acceleration voltage of 5 kV was used for appropriate excitation volumes. For the depth resolved measurements the voltage was varied from 3 kV to 7 kV.

TRCL measurements were taken at 80 K using an Attolight Allalin 4027 Chronos CL-SEM. The CL spectra and images were recorded with an iHR320 spectrometer with a focal length of 320 mm with a 150 lines/mm grating through a 700 μm entrance slit and an Andor 1024 pixel charge-coupled device. Time resolved CL spectra were recorded by utilising a laser pulsed electron gun. The laser was a frequency tripled Nd:YAG with a pulse length of 7 ps and a separation of 25 ns. A Hamamatsu streak camera was used for recording the streak images. All TRCL measurements were performed at 5 kV and a beam current of 100 pA for pulsed measurements.

For nanoprobing measurements we employed a pair of Kleindiek mm3a nanomanipulators equipped with oxide-free tungsten tips to ensure minimal contact barriers as we measured the electrical properties of the rods. Our system has triaxial connections from probe to measurement units keeping noise to a minimum. For *I*-*V* measurements the probe outputs were fed into a Keithley 236 source measurement unit. EBIC measurements instead had the outputs going into a Stanford Instruments SR570 current amplifier.

Transmission Electron Microscopy

We prepared cross-sectional specimens using slightly modified standard FIB (FEI Helios NanoLabTM) preparation techniques for STEM samples. In brief, samples were initially protected with a Pt coating ensuring sufficient Pt to fill region between the nanorods. After careful selection of the region of interest the lamella was prepared using a FIB employing standard in situ lift-out methods and adhered to a three post lift-out Cu grid via Pt deposition. Sequential FIB cleaning milling at an acceleration voltage of 30 kV was used to mill down to an $\approx$10 nm thick lamella, ensuring Pt infill via Pt electron beam between nanorods at lamella thickness of $\approx$500 nm just before the final polishing step, followed by a 5 kV treatment to reduce ion damage. The final specimen thickness was 95 nm to 120 nm, based on an electron energy loss spectroscopy measurement. A FEI Tecnai Osiris, operating at 200 kV, with a beam current of 80 pA and a high angle annular dark field (HAADF) collection semi-angle range of 65 mrad to 200 mrad was used for the structural analysis. Compositional characterisation of this sample was done using a SuperX EDS system. EDS analysis was conducted using Hyperspy and quantification determined using Cliff-Lorimer factors after background removal and peak fitting.

Electrical Contact Formation

In a clearing among the nanorod forest, the common *n*-contact was fabricated by milling down into the "substrate" by approximately 750 nm over a 1 μm^2 region and then depositing 500 nm of Pt via Ga beam deposition. A further slightly deeper trench was then etched around the contact to prevent shorting.

Nanorod *p*-contacts were made using two different methods. For "single" rod contacts we trialled both e-beam and Ga-beam deposition with a beam current of 0.48 nA. For the block contact, we first cleaved an array of rods just below the hexagonal pyramid to expose the non-polar junctions. Ga beam deposition was then used to infill the array with Pt.

Over multiple measurements over multiple weeks, we found the quality of the contact degraded significantly due to interaction with the air and the relatively low purity of the "Pt" deposited by FIB. Vacuum storage was found to prevent such degradation and samples could be measured multiple times.

As mentioned in the main text the FIB deposition was found to deteriorate the QW luminescence and for this reason electroluminescence measurements using our single wire contacting scheme were not possible. Both the e-beam and Ga-beam deposition decreased CL emission, with Ga-beam being the worse of the two, quenching all luminescence presumably due to implantation damage.

---

[1] P.-M. Coulon, B. Damilano, B. Alloing, P. Chausse, S. Walde, J. Enslin, R. Armstrong, S. Vézian, S. Hagedorn, T. Wernicke, *et al.*, Displacement Talbot lithography for nano-engineering of III-nitride materials, Microsystems & nanoengineering **5**, 1 (2019).

[2] P. R. Edwards, L. K. Jagadamma, J. Bruckbauer, C. Liu, P. Shields, D. Allsopp, T. Wang, and R. W. Martin, High-resolution cathodoluminescence hyperspectral imaging of nitride nanostructures, Microscopy and Microanalysis **18**, 1212 (2012).
